# Tamarind Trypsin Inhibitor in Chitosan–Whey Protein Nanoparticles Reduces Fasting Blood Glucose Levels without Compromising Insulinemia: A Preclinical Study

**DOI:** 10.3390/nu11112770

**Published:** 2019-11-14

**Authors:** Lídia L. R. Matias, Rafael O. A. Costa, Thaís S. Passos, Jaluza L. C. Queiroz, Alexandre C. Serquiz, Bruna L. L. Maciel, Pedro P. A. Santos, Christina S. Camillo, Catarina Gonçalves, Isabel R. Amado, Lorenzo Pastrana, Ana H. A. Morais

**Affiliations:** 1 Nutrition Postgraduate Program, Center for Health Sciences, Federal University of Rio Grande do Norte, Natal, RN 59078-970, Brazilbrunalimamaciel@gmail.com (B.L.L.M.); 2 Biochemistry Postgraduate Program, Biosciences Center, Federal University of Rio Grande do Norte, Natal, RN 59078-970, Brazil; rafaeloliveira.nutri@gmail.com (R.O.A.C.);; 3 Department of Nutrition, Center for Health Sciences, Federal University of Rio Grande do Norte, Natal, RN 59078-970, Brazil; thais_spassos@yahoo.com.br; 4 Course of Nutrition, Center University of Rio Grande do Norte, Natal, RN 59014-545, Brazil; alexandreserquiz@gmail.com; 5 Structural and Functional Biology Postgraduate Program, Biosciences Center, Federal University of Rio Grande do Norte, Natal, RN 59078-970, Brazilcamillosc@hotmail.com (C.S.C.); 6 International Iberian Nanotechnology Laboratory, 4715-330 Braga, Portugal; catarina.goncalves@inl.int (C.G.); lorenzo.pastrana@inl.int (L.P.); 7 Department of Analytical and Food Chemistry, Faculty of Science, University of Vigo, Campus As Lagoas s/n, Ourense, 32004 Galicia, Spain

**Keywords:** *Tamarindusindica* L., encapsulation, overfeeding, digestion, fasting glucose, insulin

## Abstract

In vivo studies show the benefits of the trypsin inhibitor isolated from tamarind (*Tamarindusindica* L.) (TTI) seeds in satiety and obesity. In the present study, TTI nanoencapsulation (ECW) was performed to potentialize the effect of TTI and allow a controlled release in the stomach. The impact on glycemia, insulin, and lipid profile was evaluated in *Wistar* rats overfed with a high glycemic index diet (HGLI). Characterization of the nanoparticles and in vitro stability in simulated gastrointestinal conditions, monitored by antitrypsin activity and HPLC, was performed. ECW and empty nanoparticles (CW) were administered by gavage, using 12.5 and 10.0 mg/kg, respectively. Both nanoformulations presented a spherical shape and smooth surface, with an average diameter of 117.4 nm (24.1) for ECW and 123.9 nm (11.3) for CW. ECW maintained the antitrypsin activity (95.5%) in the gastric phase, while TTI was completely hydrolyzed. In *Wistar* rats, the nanoformulations significantly reduced glycemia and HOMA IR, and ECW increased HDL-c compared to CW (*p* < 0.05).Pancreas histopathology of animals treated with ECW suggested an onset of tissue repair. Thenanoencapsulation provided TTI protection, gradual release in the desired condition, and improvement of biochemical parameters related to carbohydrate metabolism disorders,without compromising insulinemia.

## 1. Introduction

Diets with a high glycemic index, with low intake of fruits and vegetables, and the high consumption of fat, directly influence the nutritional status leading to the development of obesity and related complications, such as diabetes mellitus (DM), further intensifying the metabolic risks [1,2,3,4]. 

Studies with molecules that have bioactive properties to combat metabolic disorders related to inadequate diets, such as overweight or obesity and associated complications, are reported. Regarding diabetes mellitus type 2 (DM2), the pharmacological resources are used when it is not possible to control the blood glucose with diets and physical exercises. Medications available for diabetes therapy include oral hypoglycemic agents and insulin [5]. 

Metabolic disordersdue to high glycemic levels may also be associated with the presence of other factors, such as insulin resistance (IR) and, in this case, anti-hyperglycemic drugs are more appropriate. These drugs improve the performance of endogenous insulin, with better metabolic control [4].

Whey protein is a rich source of bioactive peptides that may play a hypoglycemic rolein the treatment of chronic diseases. These diseases arouse interest in peptides that can cross the gastrointestinal barrier with bioactive properties, modulating nervous, immune, cardiovascular, and digestive functions [6,7]. 

Chitosan is a cationic biopolymer obtained from the deacetylation of chitin, present mainly in shells of crustaceans and exoskeleton of insects [8], with several outstanding biological properties for biomedical applications [9]. Chitosan is also capable of binding to increase the stability of proteins and peptides for specific applications [10].

Protease inhibitors are proteins that reduced fasting glucose, weight, and inflammation and increased plasma CCK secretion in animal studies [11,12,13,14]. The trypsin inhibitor isolated from tamarind (*Tamarindusindica* L.) (TTI) was studied by Carvalho et al. [13], proving its satietogenic and anti-inflammatory effect in *Wistar* rats with metabolic syndrome. TTI significantly reduced triglycerides and very low-density lipoproteins (VLDL-c). Mean glucose concentration was lower in the group treated with TTI, although not significantly different from the other groups [13,14].

Thus, alternatives to potentiate the effect of TTI are needed.The encapsulation technique can improve the bioactive action, once nanoparticles (<100 nm) with uniform particle size distribution can improve the bioactive properties and promote the controlled release of bioactive molecules [15]. Queiroz et al. [16] observed that the combination ofchitosan–whey protein was essential to protect TTI and guarantee better inhibitory activity results against trypsin. Additionally, Costa [17] revealed that the same nanoformulation did not show signs of toxicity in vitro and in vivo, suggesting a safe application of these particles.

Therefore, the chitosan and isolated whey protein combination may be an interesting vehicle for biological activities when used as encapsulating agents of the tamarind isolated trypsin inhibitor (TTI). The present study aimed to evaluate the in vitro stability of nanoencapsulated TTI in simulated physiological conditions of the gastrointestinal tract and its in vivo effect on glycemia, insulin, and lipid profile of *Wistar* rats overfed with a high glycemic index diet.

## 2. Materials and Methods

### 2.1. Tamarind Trypsin Inhibitor (TTI) Extraction and Quantification

The tamarind fruit was obtained and botanically identified by the Brazilian Institute of the Environment and Renewable Natural Resources (IBAMA) seed bank in Natal-RN, Brazil, and registered in the National Genetic Heritage Management System and Associated Traditional Knowledge under number AF6CE9C.

The tamarind trypsin inhibitor (TTI) was extracted from seeds, according to Carvalho et al. [13]. The degree of purity and molecular mass of the protein isolate was verified by denaturing, discontinuous polyacrylamide gel electrophoresis (SDS-PAGE) [18]. Total protein was quantified by the Bradford method [19]. Antitrypsin activity was expressed in percentage (%) and specific activity (IU/mg protein). Trypsin inhibition assays were performed by using aliquots of 10 μL (3 μg) of trypsin and BApNa 1.25 Mm (Nbenzoyl-dl-arginine-p-nitroanilide) as substrate, according to the method proposed by Kakade [20].

### 2.2. Synthesis and Characterization of Nanoparticles

TTI nanoencapsulation was performed via thenanoprecipitation technique reported by Queiroz et al. [18], using a 1:2:2 (*w*/*w*/*w*) ratio of TTI/purified chitosan/isolated whey protein (ECW). Empty nanoparticles (CW) without TTI were also prepared, using a 1:1 (*w*/*w*) ratio of encapsulating agents. The chitosan purification step was performed based on Kumari et al. [21] with modifications proposed by Queiroz et al. [16].

Both nanoformulations (ECW and CW) were characterized by scanning electron microscopy (SEM), laser diffraction (LD), and Fourier transform infrared spectroscopy (FTIR), according to Queiroz et al. [16], with few modifications in LD. To measure the diameter and the polydispersity index (PI), the dispersions containing the nanoparticles, previously crosslinked in triplicate, were filtered, and the precipitates were dispersed in 4 mL of water and analyzed in a NanoBrookZetaPlus Zeta Potential Analyzer.

### 2.3. The Half Maximal Inhibitory Concentration (*IC50*) and Incorporation Efficiency of TTI in Nanoparticles (%)

The incorporation efficiency of TTI was determined to measure antitrypsin activity according to the methodology proposed by Kakade [20] and based on Queiroz et al. [16].

Initially, trypsin inhibition (%) was determined using 0.2, 0.4, 0.7, 1.1, 1.4, and 2.8 mg of TTI. The percent of inhibition obtained at each concentration was used to obtain IC 50 by probability test [IBM SPSS Statistic 20 software, São Paulo (SP), São Paulo, Brazil] using probity regression, with the TTI concentrations transformed to log 10 base and the response frequency as the percent inhibition. Therefore, the amount of ECW used was proportional to its composition of 1:4, and the amounts (2.1, 3.5, 5.5, 7.0, and 14.0 mg) were used to the determination of IC50. The evaluation was also performed in the CW and encapsulating agents (purified chitosan and isolated whey protein).

Thus, after obtaining these data, the efficiency of incorporation was determined, according to Queiroz et al. [16], based on the following formula [20]: encapsulation efficiency (%) = (inhibitor in the particles/inhibitor total used) × 100.

### 2.4. In Vitro Stability in Simulated Physiological Conditions of the Gastrointestinal Tract

The formulations prepared were tested under simulated oral, gastric, and intestinal conditions, in order to evaluate their stability to digestive conditions. Simulate fluids that mimic the physiological environment of the mouth (SSF), stomach (SGF), and intestine (SIF) were prepared [22]. The stock electrolytic solutions were formulated, as shown in Table 1. SSF, SGF, and SIF were prepared 1.25 times concentrated, considering the later dilution (4:1) with enzymes and CaCl_2_(H_2_O)_2_ added on the day of use, to avoid precipitation. Triplicates were performed for each condition. The assay was accomplished by using TTI (30 mg/mL), ECW (150 mg/mL), CW (120 mg/mL), and distilled water as a control.

ECW and CW were used as positive controls, without subjection to the physiological conditions of the gastrointestinal tract. The following concentrations were used in the evaluated phases: ECW in the oral phase—75 mg/mL, in the gastric phase—50 mg/mL and, in the intestinal phase—20 mg/mL; CW in the oral phase—60 mg/mL, in the gastric phase—40 mg/mL, and in the intestinal phase—16 mg/mL. This standardization was performed with consideration to the concentrations of ECW and CW submitted to physiological conditions.

#### 2.4.1. Oral

Five milliliters of sample was added to 4.0 mL of SSF, 25 μL CaCl_2_(H_2_O)_2_, and 975 mL of Milli-Q water and incubated in a thermomixer (Eddingtons Mincer Pro, 86002, Berkshire, UK), at 500 rpm (37 °C), for 10 min.

#### 2.4.2. Gastric

The gastric phase was prepared by adding to the previous mixture 7.5 mL of lecithin (10 mg/mL in SGF), 1.6 mL of pepsin (10 mg/mL in SGF), 5 μL of CaCl_2_(H_2_O)_2_, 200 μLof HCl 1 mM, and 0.695 mL of Milli-Q water. The pH was adjusted to 3.0 (using HCl 1 mM) and left in constant agitation in the thermomixer, at 500 rpm (37 °C), for 2 h.

#### 2.4.3. Intestinal

The intestinal phase was prepared by adding to the previous mixture 11 mL of simulated intestinal fluid (SIF), 5.0 mL of pancreatin (70 mg/mL in SIF), 2.5 mL of bile salts (40 mg/mL in SIF), 40 μL of CaCl_2_(H_2_O)_2_, 150 μLNaOH, and 1.31 mL of Milli-Q water. The pH was adjusted to 7.0 (using NaOH 1 mM) and left in constant agitation in the thermomixer, at 500 rpm (37 °C), for 2 h, and then the samples were centrifuged at 14,000× *g*.

After each phase, 2 mL of sample was withdrawn for follow-up analyses. All aliquots were evaluated for antitrypsin activity [20]. Chromatographic profile was evaluated via HPLC, using the Agilent 1200 Series System (Agilente, Waldbroonn, Baden-Württemberg, Germany) equipped with an Aeris TM Peptide XB-C18 column (150 mm × 2.1 mm × 3.6 μm, Phenomenex, Torrance, CA, USA). The mobile phase A was composed of water containing 0.1% trifluoroacetic acid (TFA). Phase B was composed of 60% acetonitrile with 0.1% TFA. The TTI aliquots were eluted and monitored at 280 nm by a diode array detector (DAD), using a gradient of 5% to 95% of phase B in 30 min. The flow rate was 0.4 mL/min.

### 2.5. Effect of ECW on Biochemical Parameters (Lipid Profile, Glycemia, Insulin, HOMA—IR, and HOMA-β) in Wistar Rats Overfed with HGLI Diet

#### 2.5.1. Preclinical Studies

Male *Wistar* rats were obtained from Potiguar University (UnP), kept at room temperature (25 °C), and exposed to light for 12 h. During the course of 17 weeks, the animals were overfed with an experimental diet of the high glycemic index (77.6) and glycemic load (38.8) (HGLI diet), according to Carvalho et al. [13].

After that, the rats were divided into four groups (*n* = 5), and they were acclimated and adapted to gavage for five days. During this period, the animals were fed as described in Table 2, with only water given per gavage. Then, animals were submitted to ten days of experiment, and they were fed as described in Table 2. During the ten days of the experiment, the standard diet (Labina^®^), nutritionally adequate, was also used for one of the groups of animals previously overfed for 17 weeks with an HGLI diet. The experimental diet HGLI [13] was used in the other three groups of animals. On the 11th day of the experiment, the animals were sacrificed for blood and pancreas collection to biochemical and microscopic analysis.

The concentration of ECW was established according to Costa [17]. The experiment was approved by the Ethics Committee for the Use of Animals (CEUA) and performed at Potiguar University, with protocol number 019/2017, following the recommendations of the Guide for the Care and Use of Laboratory Animals [23].

#### 2.5.2. Biochemical Parameters

The rats were fasted for 8 h and then anesthetized with 250 mg of Zolazepam Hydrochloride and 250 mg of Tiletamine Hydrochloride. Two milliliters of blood was collected by cardiac puncture, and then the animals were euthanized.

Their blood was used to determine fasting glucose, insulin, triglycerides (TG), total cholesterol (TC), VLDL-c, HDL-c, and LDL-c. Automated colorimetric–enzymatic (Labtest^®^, Natal, RN, Brazil) was used. Insulin dosage was performed by using the Rat/Mouse Insulin Elisa kit (Millipore EZRMI-13K).

To measure insulin resistance (IR) and pancreatic *β*-cell function, the Homeostasis Model (Homeostasis Model Assessment of Insulin Resistance and *β*-cells—HOMA-IR and HOMA *β*, respectively) was used based on the formulas (1) proposed by Matthews et al. [24]: HOMA-IR = (fasting insulinemia [μU/L] × fasting glucose [mg/dL]; and HOMA *β* = (20 × Insulin μIU/mL)/(Glucose mg/dL − 3.5). Table 3 presents the reference values for the biochemical parameters studied in adult male *Wistar* ratswith normal nutritional status (320–380 g), acclimated under the same conditions of the experiment, consuming Labina^®^ diet. All values used were in agreement with Guimarães et al. [25].

#### 2.5.3. Microscopic Analysis of the Pancreas

The analysis was performed according to Martins et al. [26], with some modifications. The pancreas was uniformly and systematically cross-sectioned into five parts, which were stained with hematoxylin and eosin (HE). For the microscopic analysis, the diagnostic reading of the slides was performed, with emphasis on the histological organization (20×/200 μm).

### 2.6. Statistical Analysis

The sample size was calculated, considering a simple and random sampling (Cochran model) and according to the 3Rs principle. A physiologically significant difference of the parameters evaluated was assumed when the treatment exerted a biological effect of 25% or more in relation to the group that did not receive the treatment. Furthermore, the coefficient of variation was adopted, with a probability of error <5% and power of 90%, resulting in *n* = 4.36 animals, that is, five animals per group. Data were analyzed for normality, using the Kolmogorov–Smirnov test. The Kruskal–Wallis test was necessary to verify if total cholesterol differed between the groups, using Dunn’s posthoc test to detect significant differences (*p* < 0.05). The other study variables were considered parametric and were evaluated by ANOVA and Tukey’s posthoc test, in order to determine significant differences between groups (*p* < 0.05).

## 3. Results

### 3.1. Tamarind Trypsin Inhibitor (TTI) Extraction and Quantification

**Figure 1:**
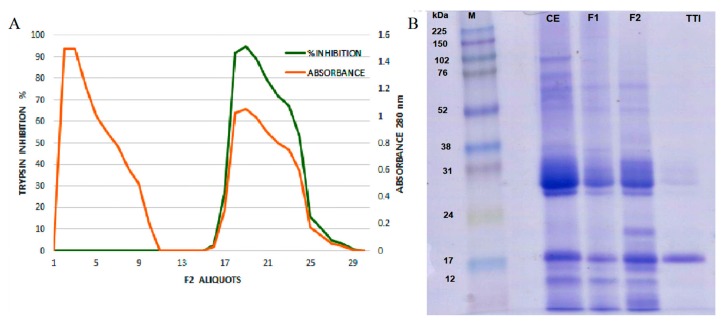
(**A**) F2 trypsin–sepharoseCNBr 4B affinity chromatographic profile of tamarind seeds by chromatography. Approximately 35 mg of F2 was applied. The column was pre-equilibrated with 50 mM of Tris-HCl buffer, pH 7.5, and the elution of unretrieved material with low antitrypsin activity (red line) was performed with the same buffer. Proteins adsorbed on the matrix (green line) were eluted with HCl (5 mM), and the protein fractions (5.0 mL) were monitored at 280 nm. The inhibitory activity of the eluted protein peak was verified by trypsin inhibition assay, using 100 μL of ITT. (**B**) Image of 12.5% SDS polyacrylamide gel electrophoresis stained with Coomassie Blue. M: Marker; CE: Crude Extract; F1: Protein fraction 1 (saturation with 0–30% of ammonium sulfate); F2: Protein fraction 2 (saturation with 30–60% of ammonium sulfate); TTI: trypsin inhibitor isolated from tamarind seeds.

TTI isolated by affinity chromatography in trypsin–sepharose (Figure 1A) was evaluated for its antitrypsin activity, and 1.4 mg of TTI promoted 100% (638 IU/mg) inhibition against trypsin. SDS-PAGE gel (Figure 1B) demonstrated the isolation of TTI, with an estimated molecular mass of 20 kDa.

### 3.2. Characterization of the Nanoformulations Obtained (CW and ECW)

**Figure 2:**
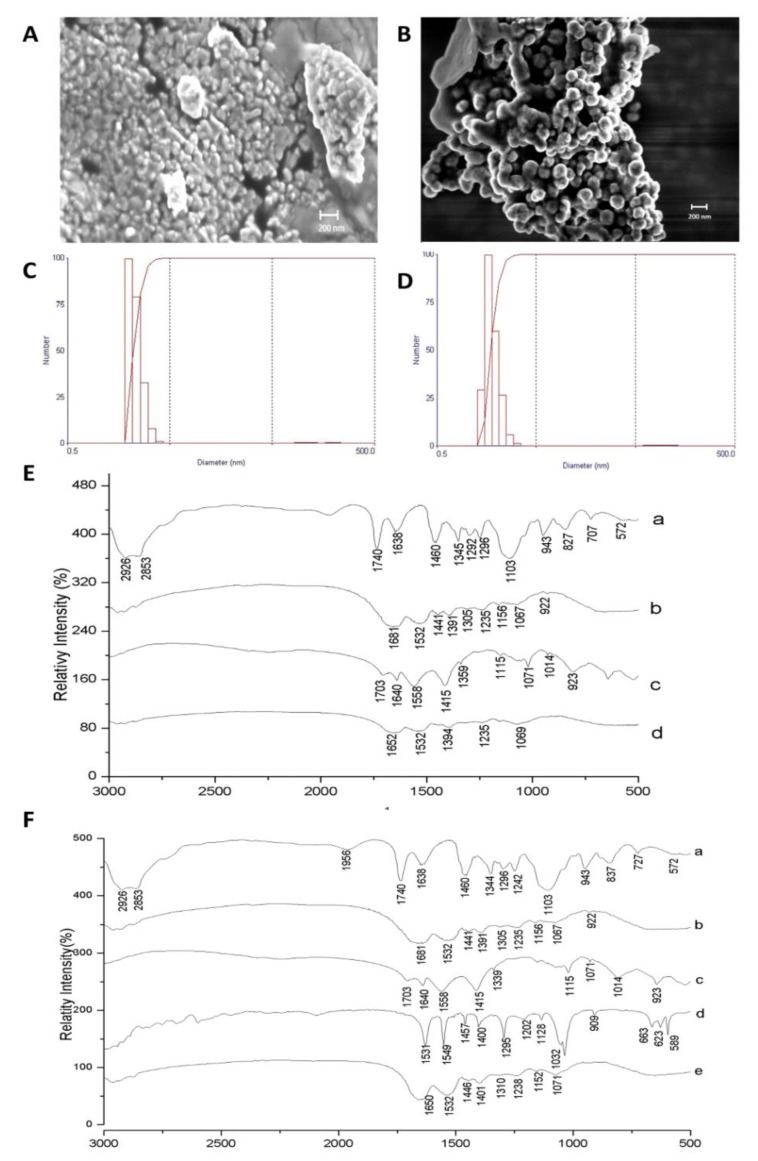
Characterization of the CW and ECW nanoformulations obtained by the nanoprecipitation technique. (**A**) Scanning electron microscopy (SEM) of CW with a magnitude of 20.00 KX. (**B**) Scanning electron microscopy (SEM) of ECW with a magnification of 20.00 KX. (**C**) Laser diffraction obtained for CW by the dispersion of nanoparticles in water and previous crosslinking by using formaldehyde. (**D**) Laser diffraction obtained for ECW by the dispersion of nanoparticles in water and previous crosslinking by using formaldehyde. Fourier transform infrared spectra (FTIR): (**E**) CW: (a) Tween 80; (b) isolated whey protein; (c) purified chitosan (d) CW: purified chitosan and isolated whey protein (1:1 *w*/*w*). (**F**) ECW: (a) tween 80; (b) isolated whey protein; (c) purified chitosan (d) tamarind trypsin inhibitor (ITT) (e) ECW: ITT encapsulated in purified chitosan and whey protein isolate (1:2:2 *w*/*w*/*w*).

Figure 2 shows the characterization results by SEM, laser diffraction, and FTIR for CW and ECW. According to the micrographs, both formulations (Figure 2A,B) presented intact and agglomerated particles with smooth surfaces, a spherical shape, and a physical size of approximately 100 nm. The mean diameter and the polydispersity index were 123.9 (11.30) nm and 0.23 (0.01) for CW (Figure 2C), and of 117.4 (24.10) nm and 0.37 (0.004) for ECW, respectively (Figure 2D).

Figure 2E,F shows the spectra obtained for CW and ECW, respectively. The bands corresponding to TTI (d), 1457 cm^−1^, and 1400 cm^−1^ (folding of the hydroxyl groups –OH) are displaced in the ECW (e) (1446 cm^−1^ and 1401 cm^−1^), and with the attenuated stretches, an indication that the encapsulating agents are protecting the active agent. Furthermore, the presence of new vibrational bands formed in the region of 1071 cm^−1^ (Figure 2F) and 1069 cm^−1^ (Figure 2E), which indicate the presence of carbon–oxygen (CO) binding between the encapsulating agents in the nanoformulations.

### 3.3. Encapsulation Incorporation Efficiency

Incorporation efficiencies were 96.0% (0.80) for ECW, while the other materials analyzed (CW, purified chitosan and isolated whey protein) did not present antitrypsin activity.

**Figure 3:**
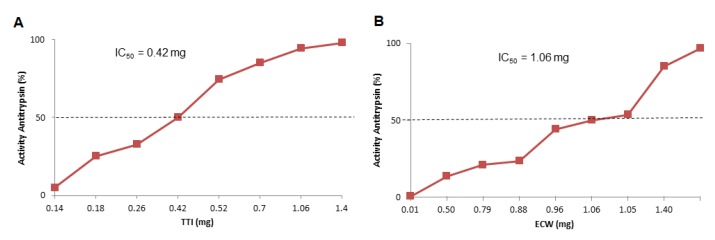
Half maximal inhibitory concentration (IC50) of antitrypsin activity estimated by IBM SPSS Statistic 20 and determined from increasing amounts of (**A**) TTI—0.2, 0.4, 0.7, 1.1,and 1.4 mg; (**B**) ECW 2.1, 3.5, 5.5, 7.0, and 14.0 mg of TTI. The inhibitory activity of the different concentrations was verified by trypsin inhibition assay, using 100 μL of TTI and ECW. TTI: trypsin inhibitor isolated from tamarind seeds; ECW: TTI encapsulated with isolated whey protein and purified chitosan (1:2:2 *w*/*w*/*w*).

TTI presented in ECW showed better antitrypsin activity, considering the proportionality between TTI, purified chitosan, and isolated whey protein (1:2:2 *w*/*w*/*w*) (Figure 3). ECW presented the same activity, with less TTI, 0.21 mg, which was half the value to obtain the same activity when compared to the isolated TTI (0.42 mg), as shown in Figure 3A.

### 3.4. In Vitro Stability in Simulated Physiological Conditions of the Gastrointestinal Tract

**Figure 4:**
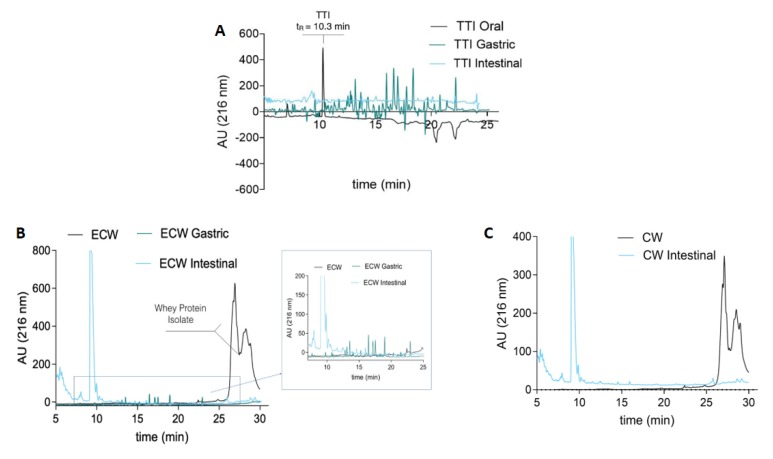
(**A**) Chromatographic profile of TTI digestion products in the oral, gastric, and intestinal phases by high-performance liquid chromatography (HPLC); 50 μL aliquots were applied. (**B**) Chromatographic profile of the ECW digestion products in the gastric and intestinal phases and (**C**) CW in the intestinal phase by high-performance liquid chromatography (HPLC); 50 μL aliquots were applied. The Agilent 1200 Series System (Agilente, Waldbroonn,Karlsruhe, Germany) chromatograph equipped with an Aeris TM Peptide XB-C18 (150 mm × 2.1 mm × 3.6 μm, Phenomenex, Torrance,CA, USA) column was used. The mobile phase A was composed of water containing 0.1% trifluoroacetic acid (TFA). Phase B was composed of 60% acetonitrile with 0.1% TFA. The TTI was eluted and monitored at 280 nm by a DAD (diode array detector), using a gradient of 5% to 95% of phase B in 30 min. The flow rate was 0.4 mL/min.

The aliquots of TTI (Figure 4A), ECW, and CW (Figure 4B,C) were also evaluated via HPLC. Table 4 shows the percentage of antitrypsin activity for TTI and ECW in each phase of the digestive process. CW did not present inhibitory activity at all stages of digestion.

After the oral phase, TTI and ECW maintained their antitrypsin activity, showing resistance to hydrolysis by oral phase enzymes. However, after the gastric phase, only ECW maintained the inhibitory activity, demonstrating the susceptibility of TTI to digestion conditions and, consequently, to gastric enzymes. The antitrypsin activity of the intestinal phase was evaluated in controls of TTI and ECW, in the same concentrations used in the digestion phases. These controls were not submitted to digestion. The antitrypsin activity of the intestinal phase for TTI and ECW was not evaluated, due to the presence of trypsin, which is the target TTI’s inhibitory activity.

The chromatographic profile of TTI in the oral phase shows a protein peak at 10.3 min, corresponding to TTI. However, this peak disappears in the gastric and intestinal phases (Figure 4A) and, in turn, several peptides appear as a reflection of the TTI hydrolysis by the enzymes involved in the digestion process. In the same way, the chromatographic profile of ECW showed a group of intense peaks at retention times between 25 and 30 min, corresponding to whey protein isolate in the capsule. These peaks disappeared after gastrointestinal digestion and led to extensive protein hydrolysis producing a mixture of peptides.

### 3.5. Evaluation of In Vivo Biochemical Parameters

**Figure 5:**
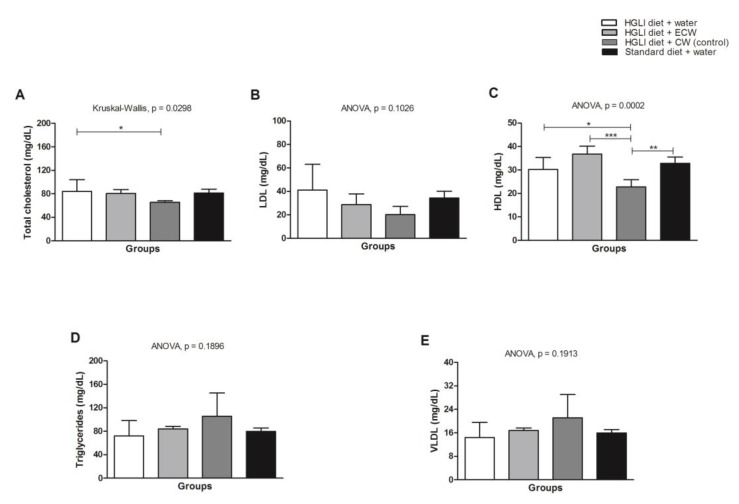
(**A**) Lipid profile in *Wistar* rats overfed with an HGLI diet and submitted to different treatments for 10 days. (**B**) HGLI diet (HGLI diet + 1 mL of water per gavage); (**C**) ECW + HGLI diet (HGLI diet + 1 mL of ECW at 12.5 mg/kg per gavage); (**D**) CW + HGLI diet (HGLI diet + 1 mL of CW at 10.0 mg/kg per gavage); (**E**) standard diet (Labina^®^ diet + 1 mL water per gavage). The coefficient of variation was adopted, with a probability of error <5% and power of 90%, resulting in all groups represent experiments with five animals. The data were evaluated for normality by Kolmogorov–Sminov (*p* > 0.05), and the statistical difference was tested by ANOVA and Tukey’s post-test (*p* < 0.05) for LDL-c, HDL-c, Triglycerides, and VLDL-c, and by Kruskal–Wallis and Dunns post-test (*p* < 0.05) for total cholesterol. Statistical comparison between the evaluated groups * *p* < 0.05, ** *p* < 0.01, and *** *p* < 0.001. HDL-C: high-density lipoprotein. LDL-C: low-density lipoprotein. VLDL-C: verylow-density lipoprotein. HGLI: high glycemic index diet and high load. ECW: trypsin inhibitor isolated from tamarind seeds encapsulated with whey protein and chitosan (1:2:2 *w*/*w*/*w*). CW: chitosan–whey protein isolated nanoparticles (2:2 *w*/*w*).

HDL-c increased in the animals overfed with the HGLI diet when compared to the CW group (Figure 5C). There was no statistical difference between the treated groups concerning LDL-c, triglyceride and VLDL-c parameters (Figure 5B,D,E).

**Figure 6:**
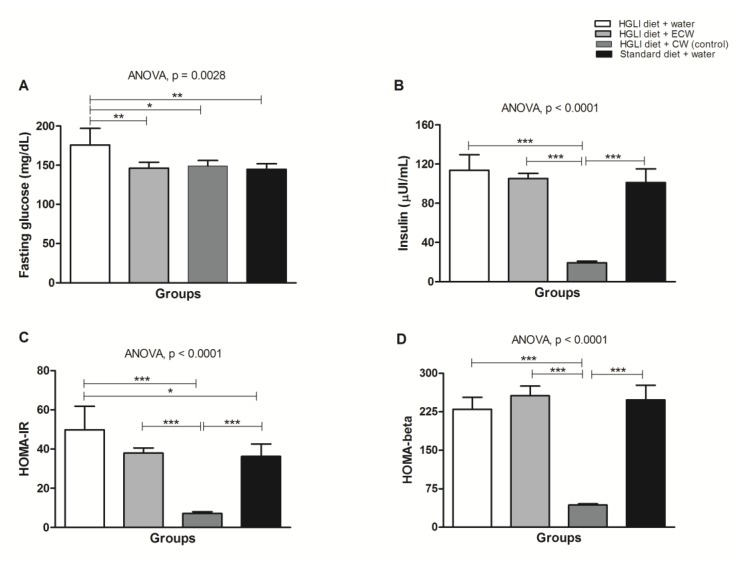
(**A**) Biochemical parameters in *Wistar* rats overfed with an HGLI diet (HGLI diet + 1 mL of water per gavage); (**B**) ECW + HGLI diet (HGLI diet + 1 mL of ECW at 12.5 mg/kg per gavage), (**C**) empty nanoparticles (CW) + HGLI diet (HGLI diet + 1 mL of CW at 10.0 mg/kg per gavage); (**D**) Standard diet (diet Labina^®^ + 1 mL of water per gavage). The coefficient of variation was adopted, with a probability of error <5% and power of 90%, resulting in all groups represent experiments with five animals. The data were evaluated for normality by Kolmogorov–Sminov (*p* > 0.05), and the statistical difference within groups was tested through ANOVA and Tukey’s post-test (*p* < 0.05). * *p* < 0.05, ** *p* < 0.01, and *** *p* < 0.01. HOMA-IR: Homeostasis Model of Insulin Resistance Homeostatic Model Assessment of Insulin Resistance. HGLI: high glycemic index diet and high load. ECW: trypsin inhibitor isolated from tamarind seeds encapsulated in chitosan purified and isolated whey protein (1:2:2 *w*/*w*/*w*). CW: chitosan-whey protein isolated nanoparticles (2:2 *w*/*w*).

Figure 6A shows that all treatments were able to significantly reduce fasting glucose of the *Wistar* rats overfed with the HGLI diet when compared to the untreated animals (*p* < 0.05). Lower concentrations of TTI in ECW were able to significantly reduce fasting glucose. CW also significantly reduced insulin, HOMA IR, and HOMA-*β* (*p* < 0.05).

### 3.6. Pancreas Histopathology of Wistar Rats

**Figure 7:**
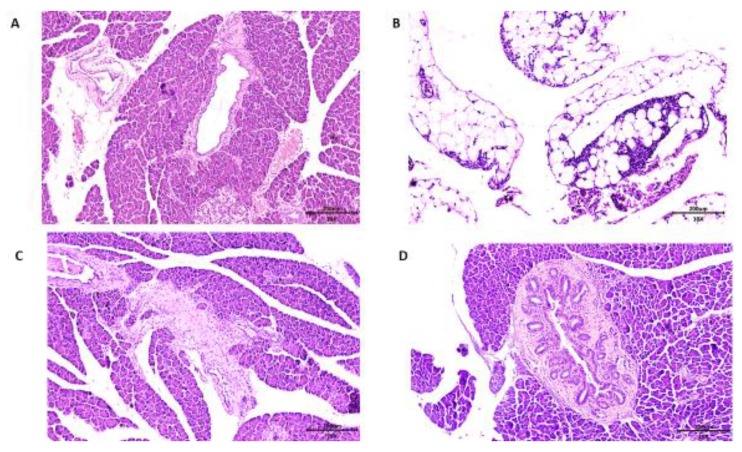
Microscopy of the pancreas histopathology in Hematoxylin and Eosin (HE) staining, comparing the effect of different treatments on overfed rats. (**A**) The HGLI diet (high index and glycemic load), (20×/200 μm) induced hyperemia, scarce inflammatory infiltrate in septa, focal areas of acinar atrophy, and focal destruction of acini; (**B**) the standard diet (Labina^®^) (10×/200 μm) induced hyperemia, focal regions of exocrine glandular parenchyma reduction, and replacement of adipose tissue; (**C**) the HGLI diet + ECW (nanoparticles based on isolated tamarind trypsin inhibitor (TTI), purified chitosan and isolated whey protein (1:2:2 *w*/*w*/*w*)) (10×/200 μm) induced mild acinar atrophy, adipose tissue unilocular in the periphery of the gland with aspect of normality; (**D**) the HGLI + CW diet (nanoparticles based on purified chitosan and isolated whey protein (1:1 *w*/*w*)) (10×/200 μm) induced hyperemia, atrophy and acinar destruction, intralobular fibrosis, and morphological pancreatic duct evidencing the appearance of numerous mural ducts.

Concerning the histopathological analysis of the pancreas, *Wistar* rats of all groups presented chronic pancreatitis. In the group of rats that received no treatment, consuming only the HGLI diet, the main changes observed were related to hyperemia and acinar destruction, as well as the presentation of inflammatory, infiltrate in the pancreatic islet (Figure 7A). In the group that received the standard diet as treatment, the same conditions were presented. However, it was possible to observe a succinct replacement of adipose tissue, indicating an onset of tissue repair (Figure 7B).

In the group that received ECW, the pancreas presented hyperemia, and a slight adipose tissue replacement in the exocrine glandular stroma parenchyma, with the presence of adipose tissue and a general aspect of normality, also suggesting an onset of tissue repair (Figure 7C). On the CW group, the pancreas presented a degenerative state, with pancreatic islet hyperplasia and morphological changes in the pancreatic ducts (Figure 7D).

## 4. Discussion

The isolation of trypsin inhibitors in tamarind seeds was reported in some studies [12,13,27], and the biochemical characteristics presented in the present study corroborate with these previous studies. The characterization of the synthesized nanoformulations was evaluated according to Queiroz et al. [16]. The results obtained in this study confirmed the homogeneous size distribution of nanoparticles.

The chemical characteristics observed show that the encapsulating agents and TTI are interacting in both formulations (ECW and CW). These characteristics associated with the observed attenuated stretches of TTI also indicate the entrapment of TTI in ECW. CW was characterized to ensure that a nanoformulation with similar physical and chemical characteristics to the ECW would be administered in rats, for comparison between the results.

The behavior or susceptibility of TTI, ECW, and CW under digestive conditions (enzymes, salts, pH, temperature, and digestion time) was performed in the in vitro stability assessment in conditions simulating the gastrointestinal tract. Three phases were considered: oral, gastric, and intestinal. TTI, ECW, and CW were monitored at each stage of the digestive process, according to Pagels and Prud’homme [28] and EFSA (European Food Safety Authority) [29].

In view of the ECW susceptibility in acidic pH and consequent gradual release of TTI observed by Costa [17], and considering the acidic pH of the gastric phase, the high percentage of antitrypsin activity (95.5%) presented by ECW might be explained not only by the fact that TTI release occurred slowly, as demonstrated by Costa [17], but also by the predominance of TTI still interacting with chitosan particles in the analyzed gastric phase. Furthermore, the ECW concentration in the gastric phase was almost twice (50 mg/μL) the minimum level (28 mg/μL) to reach 100% antitrypsin activity, according to IC 50 (Figure 3). This result for ECW may explain the high inhibitory activity against trypsin in an acid medium (stomach), in which TTI was expected to be free and consequently completely digested.

Due to the interaction of TTI with the encapsulating agents, visualization in HPLC would be unlikely, as observed (Figure 1B), once TTI acquired a new conformation through nanoencapsulation. Chanphai and Tajmir-Riahi [30,31] also observed a significant alteration of the soybean trypsin inhibitor conformation when interacting with chitosan through Van der Waals forces.

Since chitosan is a polysaccharide, the inability to visualize the protein peaks in HPLC corresponding to TTI in the intestinal phase reinforces the hypothesis that TTI present in ECW is strongly interacting with chitosan, as observed by Costa [17].

The results indicate that, in the intestinal phase, there was a mixture of released TTI, and TTI interacted with chitosan particles. This mixture was conditioned by acid pH, which promoted the digestion of the isolated whey protein in the stomach phase (Figure 1B). The same process occurs with CW, as shown in Figure 1C, and, in the intestinal phase, chitosan particles are observed without the outer coating of isolated whey protein. These results were confirmed in chromatograms by the absence of protein peaks in the intestinal phase. These peaks were observed in the oral phase, indicating the presence of isolated whey protein. Therefore, nanoencapsulation was effective in protecting and releasing TTI in the desired condition, so it was extremely important to evaluate the in vivo effects related to the bioactive properties of nanoencapsulated TTI.

Regarding the in vivo analyses, the increase in total cholesterol in the group treated with ECW (Figure 5A) is justified by the rise of HDL-c, which is a cholesterol transporter found in the blood circulation [32].

There was no statistical difference between the treated groups for LDL-c, triglycerides, and VLDL-c (Figure 5B,D,E). Carvalho et al. [13] investigated the effect of isolated TTI on the lipid profile of *Wistar* rats with metabolic syndrome; however, no statistical difference was observed between the groups regarding HDL-c. In the present study, an increase in HDL-c was observed compared to the untreated group, although not significant (*p* > 0.05). On the other hand, the ECW-treated group significantly increased the HDL-c concentration compared to the group receiving nanoparticles without TTI (CW). This fact, associated with the nonsignificant increase in triglycerides (*p* > 0.05) promoted by CW, shows the beneficial effects supported by encapsulated TTI (ECW).

Considering the glucose results, the standard diet is considered the conventional treatment, and ECW and CW were responsible for the reduction of fasting glucose, supposedly by different mechanisms. However, ECW presented improved TTI effect through encapsulation. This improvement in effect can be evidenced by comparing our results with the study conducted by Carvalho et al. [13], which found no reduction in fasting glucose. In the present study, fasting glucose reduced significantly by using lower levels of TTI, suggesting an in vivo hypoglycemic effect due to the encapsulating process.

Costa et al. [27] suggested that TTI can act as a hormone, binding to specific receptors. This hypothesis is based on the study with a bioactive peptide (trypsin inhibitor) with hypoglycemic action observed in bitter melon, *Momordica charantia* (mcIRBP) [33]. It acts by activating the insulin-receptor signaling pathways and stimulating both the uptake and release of glucose in rat cells [34,35,36].

Another hypothesis to explain this effect could be related to the probable probiotic action of TTI, or even its digestion peptides, when reaching the large intestine, which could indirectly influence the lowering of glucose in the blood, [37] and further studies should address this indirect mechanism.

Regarding the reduction of fasting glucose concentrations in the CW group (Figure 3A), this canprobably be attributed to chitosan, which exhibits hypoglycemic activity, as observed in previous studies [38,39,40,41]. Additionally, CW also significantly reduced insulin, HOMA IR, and HOMA-*β*. Furthermore, whey protein isolate also exhibits, after digestion, high postprandial concentrations of specific amino acids that may contribute to glucose homeostasis [42].

In contrast, ECW did not change insulin, HOMA-*β*, and HOMA IR, when compared to the groups that received the HGLI diet.

Once all groups of animals were overfed, for 17 weeks, a high glycemic index diet before treatments, glucose concentrations increased as a response to the HGLI diet, which stimulated the release of insulin. Consequently, insulin resistance (IR) induced by this diet reduced insulin sensitivity from effectively metabolizing glucose in the untreated groups. HOMA-IR ranging from 2.5 to 3.9 [24,43,44] may indicate IR, and HOMA-*β* greater than 100 indicates *β*-pancreatic-cell dysfunction [24].

Considering the effect of ECW on the parameters analyzed in this study, ECW may be a candidate for the treatment of metabolic disorders as an adjuvant therapy. There are no reports of these joint effects on fasting glucose and lipid profile components for trypsin inhibitors.

In the present study, under physiological conditions simulating the gastrointestinal tract, not only TTI’s hydrolysis products from the gastric phase were in the intestinal phase, but also ECW. Thus, the bioactive effects observed in the study could be, in part, promoted by the interaction with chitosan. These results are innovative and unprecedented, considering the isolated bioactive effects of TTI and chitosan, for which previous research have used higher concentrations than those in this [45,46,47,48].

Thus, analyses that evaluate the direct cause–effect relationship of TTI or ECW and their bioactivities are necessary to understand the results regarding the improvement in biochemical parameters in overfed *Wistar* rats, as well as their physiological mechanisms. To understand the results obtained regarding glycemia and insulinemia, it is necessary to investigate other aspects, such as the pancreatic morphology. The pancreas releases several hormones, including insulin (*β*-cells) and glucagon (*α*-cells), which are both released directly from the bloodstream through a vascular network and have a relevant role in the regulation of blood glucose concentrations [49,50], factors of interest in the present study.

Alterations in the pancreas were reported in a study using the HGLI diet [51]. Morphological changes in the pancreas of diabetic rats were shown in the studies of Ragavanand Krishnakumari [52], as well as Simon et al. [53], which indicated separate lobes with lobular infiltration of lymphocytes, damaged tissue, *β*-cells degeneration, and damage of the Langerhans islets. In the present study, all groups presented chronic pancreatitis due to the HGLI diet administered in all groups of animals.

At the same time, ECW gave better results when compared to other treatments, considering that chronic pancreatitis was presented by all *Wistar* rats (Figure 4C). The CW group presented intensive degeneration of the pancreas, with pancreatic islet hyperplasia and morphological changes in the pancreatic ducts (Figure 4D) aggravating the degenerative state of the pancreas observed in all groups.According to Castro et al. [54] and Eliakim-Ikechukwu and Obri [55], the morphologicalchanges observed in the pancreas of *Wistar* rats treated with CW contribute to the reduction of insulin concentrations. Thus, the pancreatic morphology presented may be directly related to the low concentrations of insulin presented by the group of CW-treated rats (Figure 4B).

Seo et al. [39] evaluated the effects of commercial nanochitosan on rat pancreases by histological analysis. The researchers found that nanochitosan with a mean diameter of 562 nm did not promote the destruction of pancreatic *β*-cells. Therefore, we emphasize the importance of the present study, which was the first preclinical study to evaluate the effect of chitosan-based nanoparticles with an average diameter of approximately 100 nm, which promoted harmful impact on the organ evaluated, compromising insulinemia. This might be related to the increased chitosan reactivity provided by the smaller particle size. The results suggested that this effect was minimized in ECW due to the presence of TTI, which, through chemical interactions with chitosan, reduced its reactivity.

## 5. Conclusions

This study demonstrated that nanoencapsulation improved the effect of TTI onfasting-blood glucose levels. The encapsulation was accomplished by using a low TTI concentration, without worsening chronic pancreatitis presented nor compromising insulinemia by the animals studied.

## Figures and Tables

**Table 1:** Chemical composition of simulated salivary fluid (SSF), simulated gastric fluid (SGF), and simulated intestinal fluid (SIF) prepared from the stock solutions.

Chemical	Stock Solution		SSF (pH 7)		SGF (pH 3)		SIF (pH 7)	
	g/L	mol/L	mL	mmol/L	mL	mmol/L	mL	mmol/L
KCl	37.3	0.5	15.1	15.1	6.9	6.9	6.8	6.8
KH_2_PO_4_	68.0	0.5	3.7	3.7	0.9	0.9	0.8	0.8
NaHCO_3_	84.0	1.0	6.8	13.6	12.5	25.0	42.5	85.0
NaCl	117	2.0	-	-	11.8	47.2	9.6	38.4
MgCl_2_(H_2_O)_6_	30.5	0.15	0.5	0.15	0.4	0.12	1.1	0.33
(NH_4_)_2_CO_3_	48.0	0.5	0.06	0.06	0.5	0.5	-	-
CaCl_2_(H_2_O)_2_	44.1	0.3		1.5		0.15		0.6
HCl	-	6.0	0.9	1.1	13.0	15.6	0.7	8.4

**Table 2:** Groups of studied *Wistar* rats and their diets.

Groups	*N*	Treatment
1	5	HGLI Diet + 1 mL of water per gavage
2	5	HGLI Diet + 1 mL of ECW (12.5 mg/kg per gavage)
3	5	HGLI Diet + 1mL of CW (10.0 mg/kg per gavage)
4	5	Standard Diet + 1 mL of water per gavage

**Table 3:** Reference values of the biochemical parameters in theadult male *Wistar* rats of normal nutritional statusthat wereacclimatized at UnP/Brazil, in January 2019.

Parameters	Mean (SD)
Fasting blood glucose (mg/dL)	88.80 (17.87)
Insulin (µU/mL)	12.17 (0.73)
HOMA-IR	2.67 (0.63)
HOMA—BETA	47.44 (9.07)
Total cholesterol (mg/dL)	112.00 (54.00)
HDL-c (mg/dL)	23.40 (4.04)
LDL-c (mg/dL)	22.76 (4.05)
VLDL-c (mg/dL)	20.65 (5.59)
Triglycerides (mg/dL)	100.18 (29.80)

**Table 4:** In vitro antitrypsin activity (%) of TTI and ECW in simulated conditions of the gastrointestinal tract.

	Antitrypsin Activity (%)				
Aliquots	TTI Mean %	ECW Mean%	Water Mean %	TTI * Mean %	ECW * Mean%
**Control**	-	-	-	100	100
**Oral**	100	100	0.0	100	100
**Gastric**	0.0	95.5	0.0	85.0	100
**Intestinal**	-	-	0.0	50.0	90.0

* Percentage of antitrypsin activity of TTI and ECW at concentrations in the digestion phases (in water) without digestion. For antitrypsin activity, 10 μL (3 μg) of trypsin, 100 μL of TTI and ECW, and BApNa (1.25 Mm) (Nbenzoyl-dl-arginine-p-nitroanilide) were used as substrate.
